# Wearable Device–Based Respiratory Complexity Analysis for Detecting Pulmonary Congestion in Patients With Heart Failure: Observational Exploratory Study

**DOI:** 10.2196/73488

**Published:** 2025-08-27

**Authors:** Mengwei Li, Yu Kang, Shuanglin Zhao, Xiu Zhang, Zheng Liu, Lixuan Li, Qing Zhang, Zhengbo Zhang

**Affiliations:** 1 Department of Medical Engineering, Beidaihe Rest and Recuperation Center of PLA, Qinhuangdao, China; 2 Center for Artificial Intelligence in Medicine, Chinese PLA General Hospital, 28 Fuxing Road, Haidian District, Beijing, China, 86 13693321644; 3 Department of Cardiology, West China Hospital of Sichuan University, Chengdu, China; 4 Department of Medical Engineering, The 72nd Group Army Hospital of CPLA, Huzhou, China

**Keywords:** respiratory complexity, pulmonary congestion, heart failure, multiscale entropy, wearable device

## Abstract

**Background:**

Excessive pulmonary congestion (PC) is a major contributor to heart failure (HF) deterioration and often necessitates emergency hospitalization. Early detection of PC-related respiratory abnormalities via wearable devices could enable prompt intervention and reduce admissions. However, the feasibility of using wearables to detect specific respiratory biomarkers of PC remains unclear.

**Objective:**

This study aimed to evaluate the feasibility of using wearables to monitor respiratory data in hospitalized patients with HF and to determine if these signals could distinguish patients with HF and PC.

**Methods:**

This single-center, observational, exploratory study enrolled hospitalized patients with HF without severe lung diseases or need for intensive care or ventilation. All participants wore a designated device for ≥24 hours starting within 24 hours after admission; only nighttime data were analyzed. Breathing patterns were quantified across the respiratory cycle, amplitude, and multiscale entropy (MSE). Patients underwent comprehensive evaluations, including vital signs, laboratories, echocardiography, and a 28-zone lung ultrasound to define PC (>5 B-lines) within the first 24 hours.

**Results:**

The study enrolled 62 patients with HF between May 2021 and November 2022, including 44 with PC. Compared with patients with non-PC, those with PC demonstrated significantly prolonged mean expiratory time (TE; mean 2.17, SD 0.43 s vs mean 1.94, SD 0.34 s, *P*=.03), elevated expiratory time ratio (TE_ratio; mean 59.12%, SD 2.94% vs mean 56.4%, SD 3.36%, *P*=.006), and higher area under the curves (AUC) of MSE values for scales 1 to 5 (area_1_5) and scales 6 to 20 (area_6_20) in respiratory amplitude (RA; area_1_5 mean 4.20, SD 1.52 vs area_1_5 mean 2.93, SD 1.03, *P*<.001; and area_6_20 mean 8.86, SD 3.14 vs area_6_20 mean 12.28, SD 4.84, *P*=.002). Logistic regression identified mean TE_ratio, RA area_1_5, and RA area_6_20 as significant predictors of PC (*P*<.05). After adjusting for clinical confounders, both RA area_1_5 and RA area_6_20 remained independently associated with PC. Receiver operating characteristic analysis revealed that RA area_1_5 had the largest AUC of 0.75 (95% CI 0.63‐0.88, *P*=.002), with 65.9% sensitivity and 73.3% specificity. For the multivariate logistic regression model constructed using combined parameters of diastolic blood pressure, logarithmically transformed N-terminal pro-B-type natriuretic peptide, New York Heart Association class IV, mean TE_ratio, RA area_1_5, and RA area_6_20, the AUC was 0.91 (95% CI 0.84-0.98, *P*<.001), sensitivity of 77.3%, and specificity of 88.9%.

**Conclusions:**

In this exploratory study, wearable-based MSE analysis distinguished hospitalized patients with HF with and those without PC, showing prolonged expiratory phases and increased respiratory amplitude complexity in the PC group.

## Introduction

Breathing patterns refer to the characteristics of respiratory movements, mainly featured by 4 scales: the rate (frequency), depth (drive), mode of breathing (symmetry), and regularity of breathing pattern (rhythm). Each of these entities can be affected by normal physiological events and disease processes, which contribute to the breath-to-breath variations observed in health and disease [1-3]. These complex patterns not only indicate the severity of the condition but also may have prognostic implications [4]. Consequently, the respiratory pattern is a critical component of vital sign monitoring by bedside monitors during hospitalization, aiding severity assessment and therapeutic guidance [5]. More recently, wearable technologies, with their advantages of being noninvasive, independent, and capable of continuous monitoring, are emerging as a promising home-based monitoring tool for breathing pattern alteration, aiming to early diagnose or disease severity monitoring [6].

Heart failure (HF) is a common condition that can lead to significant alterations in breathing patterns. One of the primary causes of respiratory abnormalities in HF is pulmonary congestion (PC), which results from fluid accumulation in the lungs due to elevated left ventricular filling pressure [7-15]. In its mild to moderate stages, patients may experience gradual and often undetectable fluid retention, which may be asymptomatic or present with only mild dyspnea—insufficient to prompt medical attention. However, severe PC (pulmonary edema) manifests as acute dyspnea and typically requires emergency hospitalization. Beyond fluid overload, other mechanisms contribute to respiratory pattern changes in HF, including increased sympathetic activity and heightened central and peripheral chemosensitivity [16-18]. Early detection of PC and prompt decongestive therapy could serve as a key management strategy to prevent acute decompensation and hospitalization. Home-based monitoring of PC-related respiratory abnormalities may offer a promising approach for early detection of HF exacerbation. However, the specific respiratory biomarkers detectable via wearable devices—and their direct association with PC—remain unclear. To address this gap, we aimed to evaluate the feasibility of using wearable devices to monitor respiratory data in hospitalized patients with HF and to determine whether respiratory signals collected by these devices can distinguish patients with HF with PC from those without PC.

## Methods

### Study Design

This was a single-center, observational, exploratory study. We enrolled hospitalized patients aged ≥18 years with a confirmed diagnosis of HF in the Department of Cardiology, West China Hospital, Sichuan University. Patients were excluded if they met any of the following criteria: (1) severe lung diseases, such as pulmonary fibrosis, emphysema, or severe chronic obstructive pulmonary disease (COPD), as these conditions are associated with abnormal breathing patterns and could confound PC assessment via lung ultrasound (LUS); (2) clinician-determined ineligibility, including active malignancies or pregnancy, due to potential risks or study interference; (3) acute pulmonary edema or severe dyspnea requiring intensive care or mechanical ventilation, as these conditions necessitate urgent intervention and may distort respiratory pattern analysis; and (4) patient refusal to participate.

All the enrolled patients were required to wear the wearable device for at least 24 hours since the day of admission. Within the first 24 hours after admission, a comprehensive evaluation including vital signs, laboratory tests, echocardiography, and PC assessment by LUS was performed.

### Ethical Considerations

The study was approved by the Biomedical Research Ethics Committee of West China Hospital, Sichuan University (20211045). The study qualified as minimal-risk observational research involving routine clinical care augmentation. All participants provided written informed consent before enrollment. All collected data were deidentified using the following measures: clinical data were stored with pseudonymized codes, and the master linkage file is maintained separately under password protection. Participants received nonmonetary compensation as a full waiver of outpatient follow-up consultation fees (270 CNY, approximately US $43 at the exchange rate during the study period) and complimentary device-derived health reports. No direct financial payments were made to avoid undue inducement. The fee waiver mechanism was preapproved by the ethics committee as equitable compensation.

### Clinical Data Collection

Within 24 hours postadmission, the following clinical data were recorded: (1) demographic, comorbidities (diabetes, hypertension, hyperlipidemia, chronic kidney disease, and sleep disorders), and medications (Angiotensin-Converting Enzyme Inhibitors, Angiotensin II Receptor Blockers, Angiotensin Receptor-Neprilysin Inhibitors, beta-blocker, sodium-glucose cotransporter-2 inhibitors, Mineralocorticoid Receptor Antagonists, and diuretics) by history taking and verified through medical record review; (2) blood pressure, weight, height, and BMI measured by the nurse; (3) the echocardiographic data of left ventricular ejection fraction (EF); (4) New York Heart Association (NYHA) functional class, Borg score, EuroQol Five Dimensions Questionnaire assessed by researchers.

### PC Assessment by LUS Scan

LUS was performed by an experienced investigator, who was blinded to the results of clinical congestion assessment and other clinical data. Patients were asked to lie in a recumbent position during the LUS examination performed using a pocket ultrasound device (Lumify, Phillips Healthcare) with a phased array transducer in sagittal orientation at an imaging depth of 18 cm. A 28-zone protocol was used for image acquisition as previously recommended [19]. The highest number of B-lines visualized during the entire clip was quantified for each zone, and the sum of the 28 zones was used for analysis; PC(+) was defined as the presence of >5 comets [20].

### Wearable Device and Respiratory Signals Extraction

**Figure 1.**
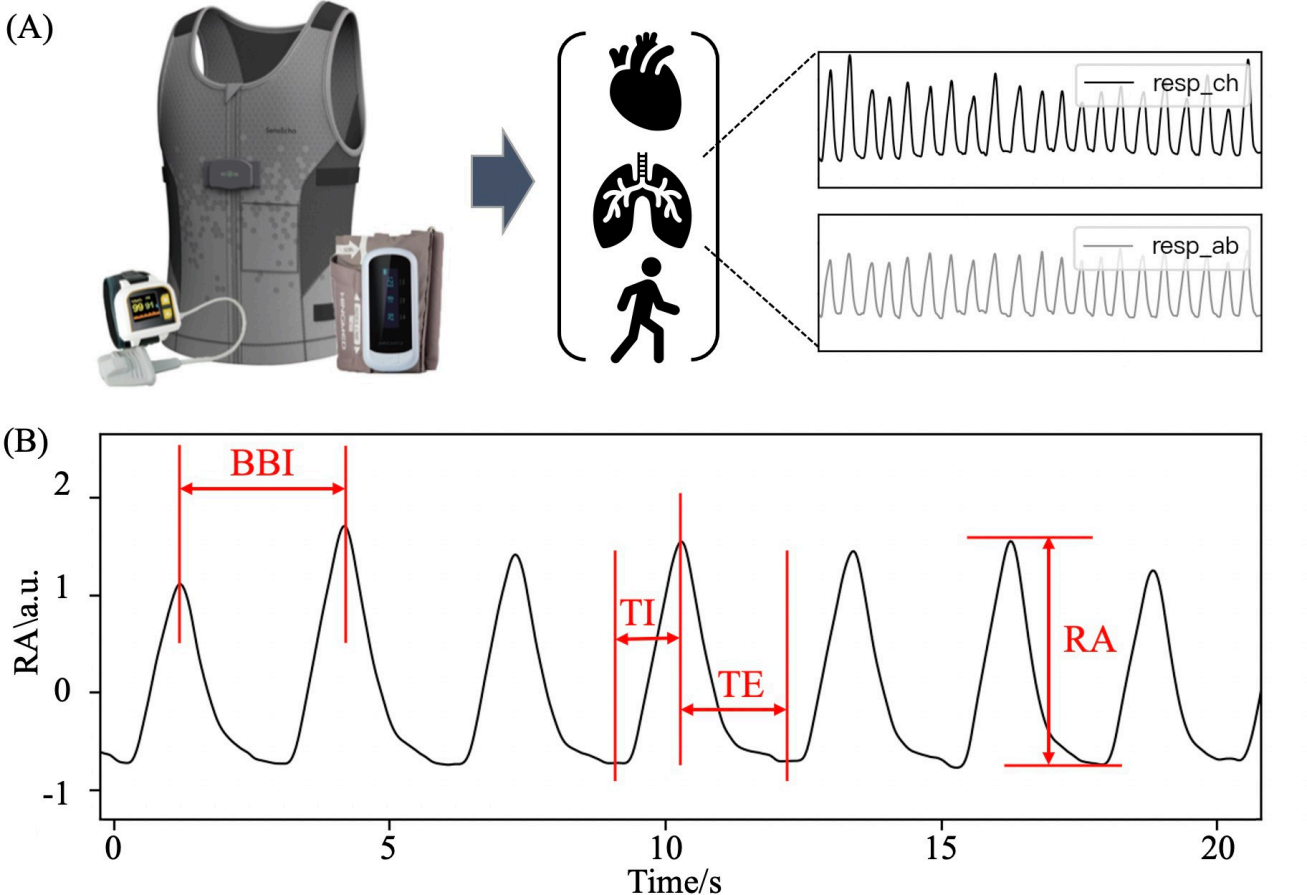
Wearable device and parts of breathing pattern indicators. (**A**) Wearable device and collected physiological signals. (**B**) Parts of breathing pattern indicators. a.u.: arbitrary units; BBI: breath-to-breath intervals; RA: respiratory amplitude; resp_ch: chest respiratory; resp_ab: abdominal respiratory; TE: expiratory time; TI: inspiratory time.

The wearable device used is the SensEcho physiological monitoring system, as illustrated in Figure 1A, which could continuously collect physiological data [3]. The device uses Respiratory Inductive Plethysmography technology to capture chest and abdominal respiratory movement signals at a sampling frequency of 25 Hz, as well as electrocardiogram signals at 200 Hz, and 3-axis accelerometer signals at 25 Hz. Chest and abdominal movement signals were collected using wearable devices for 24 hours on the day of admission. However, only signals collected at nighttime were used for analysis. Because nighttime monitoring is more comparable than daytime monitoring, as the nighttime environment generally remains consistent, involving fewer external interference factors, such as visits from relatives, medical activities, or variations in external environments [21].

Only the signals during the nighttime period (11 PM to 5 AM) were used for analysis. During nighttime, patients frequently engaged in movements such as sitting up, turning over, or adjusting clothing. To address the resulting artifacts caused by significant amplitude changes during these posture shifts, we identified movement periods using the 3-axis accelerometer signal and excluded the corresponding respiratory data. The chest respiratory signals and abdominal respiratory signals were aligned by timestamp and added together to generate the total respiratory signal. Subsequently, a linear detrending process was used to remove slow baseline drift or fluctuations and to center the total respiratory signals around zero. A fifth-order, 2 Hz low-pass infinite impulse response Butterworth filter was then used to eliminate high-frequency noise and obtain clean respiratory signals. In total, 527.19 hours of respiratory signals were included, accounting for 91.51% of the total dataset. The extremum-point-search algorithm was used to detect and identify the peaks and valleys of the respiratory wave [22], thus obtaining the breath-to-breath intervals (BBI) sequence, as well as the respiratory amplitude (RA) sequence through the calculation of distances between the peaks and valleys of each breath.

### Respiratory Signal Analysis

Based on the BBI and RA, several parameters could be derived as listed in the following. All these parameters have been validated and could discriminate patients with HF from healthy participants in our previous study [23].

#### Respiratory Cycle Parameters

The BBI obtained through breath-to-breath calculations was used to derive the breathing rate (BR) for each breath, yielding the mean BR and the coefficient of variation of BR. The inspiratory time, expiratory time (TE), and the ratio of expiratory time (TE_ratio) were calculated from the peaks and valleys, where TE_ratio represents the ratio of time taken to complete 1 exhalation to the total time taken for 1 respiratory cycle. In this study, the mean and coefficient of variation of inspiratory time, TE, and TE_ratio were calculated, with the mean reflecting the level of respiratory interval duration and the coefficient of variation reflecting the variability of the respiratory interval. Some of the parameters are illustrated in Figure 1B.

#### RA Parameters

This includes the coefficient of variation of RA, the coefficient of variation of the relative rapid-shallow-breathing index (R_RSBI), and the respiratory instability index. The R_RSBI is the ratio of BR to RA, and the respiratory instability index is the IQR of the ratio of RA to the breath interval, which is similar to the respiratory instability by Kumagai et al [8]. These parameters reflect the variability and stability of RA. The RA measured using wearable devices in this study was relative, and the actual tidal volume values could not be calculated; only the relative differences between groups could be analyzed; therefore, only the coefficient of RA was studied. Furthermore, R_RSBI is a relative value compared with itself, which is different from the traditional RSBI value.

#### Multiscale Entropy Parameters

Sample entropy (SampEn) is a probability-based entropy measurement used to assess irregularity and the predictive capability of distributions among observed samples in a given time series of length *m* [24]. Multiscale entropy (MSE) combines multiscale and nonlinear features, providing an additional observational perspective to comprehensively assess the complexity of physiological signals at different time scales, making it more flexible [25]. In this study, MSE analysis was performed on the BBI and RA time series. The analyzed parameters include the area under the curve (AUC) of MSE for scales 1 to 5 (area_1_5), the AUC for scales 6 to 20 (area_6_20), and the slope of the linearly fitted MSE curve for scales 1 to 5. MSE reflects the complexity and irregularity of respiratory sequences on different time scales.

### Statistical Analysis

Continuous variables are presented as mean (SD) or median (IQR), while categorical variables are presented as n (%). Independent sample *t* tests are used for the comparison of continuous variables with normal variance, while the Mann-Whitney *U* test is used for skewed continuous variables, and the chi-square test or Fisher exact test is used for categorical variables. Logistic regression analysis is used to identify risk factors associated with PC. Considering that age and gender may have an impact on breathing patterns [5], age, gender, and factors identified as significant in univariate regression analysis are then sequentially included in the model for adjustment to determine the corrected risk factors for PC. The receiver operating characteristic (ROC) curve is used to evaluate the ability of respiratory pattern parameters to identify HF combined with PC. A significance level of *P*<.05 was considered statistically significant. Data processing and statistical analysis were performed using Python 3.7 and IBM SPSS 25.

## Results

### Overview

A total of 62 patients with HF (mean age 60.53, SD 14.92 y, 38/62, 61% male) were enrolled between May 2021 and November 2022, including 19 (31%) patients with reduced EF and 43 (69%) with preserved EF. The comorbidities included 30 (48%) patients with hypertension, 17 (27%) with diabetes. By LUS examination, there were 44 (80%) patients with PC (PC group) and 18 (20%) patients without PC (non-PC group). By comparison, the PC group had lower diastolic blood pressure (DBP) and higher N-terminal pro-B-type natriuretic peptide than the non-PC group, while the rest of the parameters listed in Table 1 were comparable.

**Table 1.** Baseline characteristics of patients with heart failure with and those without pulmonary congestion.

Variable	non-PC^a^ (n=18)	PC^b^ (n=44)	*P* value
Age (y), mean (SD)	56.44 (14.77)	62.20 (14.82)	.17
Male, n (%)	10 (56)	28 (64)	.76
Height (m), mean (SD)	1.64 (0.07)	1.62 (0.07)	.31
Weight (kg), mean (SD)	67.74 (19.89)	59.12 (12.27)	.10
BMI (kg·m^−2^), mean (SD)	25.04 (6.42)	22.51 (3.92)	.13
Diabetes, n (%)	5 (28)	12 (27)	≥.99
Hypertension, n (%)	11 (61)	19 (43)	.32
Hyperlipidemia, n (%)	2 (11)	2 (5)	.57
CKD^c^, n (%)	15 (37)	7 (41)	.98
Sleep disorders, n (%)	2 (11)	2 (5)	.57
LVEF^d^, n (%)			.21
HFpEF^e^	4 (22)	15 (34)	
HFrEF^f^	14 (78)	29 (66)	
SBP^g^ (mm Hg), mean (SD)	125.11 (21.86)	113.84 (21.26)	.07
DBP^h^ (mm Hg), mean (SD)	82.89 (15.04)	71.57 (11.44)	.008
NYHA^i^, n (%)			.11
II	3 (17)	5 (11)	
III	10 (56)	14 (32)	
IV	5 (28)	25 (57)	
NT-proBNP^j^ (pg/mL), median (IQR)	3343.00 (1972.75-6907.25)	7406.00 (4244.50-10871.50*)*	.009
LUS B line^k^, median (IQR)	2.50 (0.00-4.00)	13.00 (9.00-21.50)	*<*.001
Borg score, n (%)			.06
0‐2	13 (81)	17 (46)	
3‐4	3 (19)	19 (51)	
≥5	0 (0)	1 (2.70)	
EQ5D_UI^l^, mean (SD)	0.79 (0.28)	0.64 (0.33)	.08
EQ5D_VAS^m^, mean (SD)	61.47 (20.45)	57.67 (20.13)	.52
Medication, n (%)			
ACEI, ARB, or ARNI^n^	10 (56)	17 (39)	.35
Beta-blocker	8 (44)	23 (52)	.78
SGLT2i^o^	4 (22)	14 (32)	.66
MRA^p^	8 (44)	18 (41)	≥.99
Diuretics	17 (94)	44 (100)	.29

a non-PC: patients with heart failure without pulmonary congestion.

b PC: patients with heart failure with pulmonary congestion.

c CKD: chronic kidney disease.

d LVEF: left ventricular ejection fraction.

e HFpEF: heart failure with preserved ejection fraction.

f HFrEF: heart failure with reduced ejection fraction.

g SBP: systolic blood pressure.

h DBP: diastolic blood pressure.

i NYHA: New York Heart Association.

j NT-proBNP: N-terminal pro-B-type natriuretic peptide.

k LUS: lung ultrasound.

l EQ5D_UI: European Quality of Life Five Dimension Questionnaire-Utility Index.

m EQ5D_VAS: European Quality of Life Five Dimension Questionnaire-Visual Analog Scale.

n ACEI/ARB/ARNI: Angiotensin-Converting Enzyme Inhibitors, Angiotensin II Receptor Blockers, or Angiotensin Receptor-Neprilysin Inhibitors.

o SGLT2i: sodium-glucose co-transporter 2 inhibitors.

p MRA: mineralocorticoid receptor antagonists.

### Respiratory Signals Comparison

**Figure 2.**
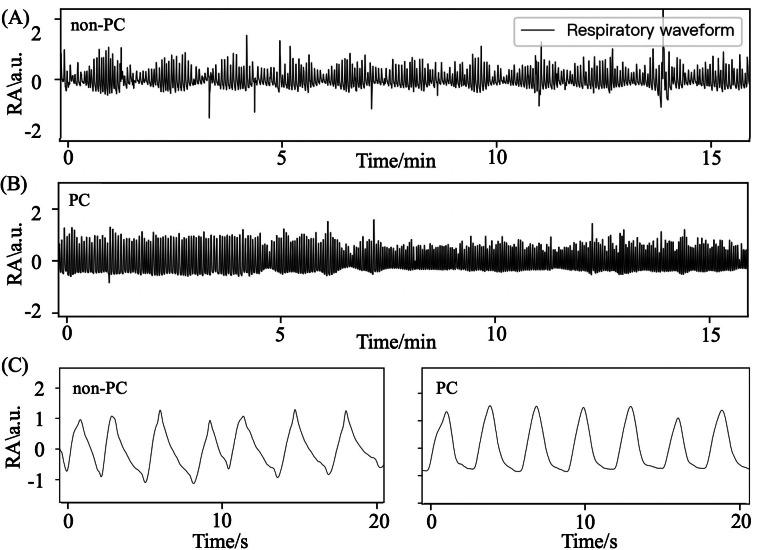
Respiratory waveforms of heart failure patients with and without pulmonary congestion. (A) Heart failure patients with pulmonary congestion and (B) without pulmonary congestion. (C) Part of the amplified respiratory wave. a.u.: arbitrary units; PC: pulmonary congestion; RA: respiratory amplitude.

**Figure 3.**
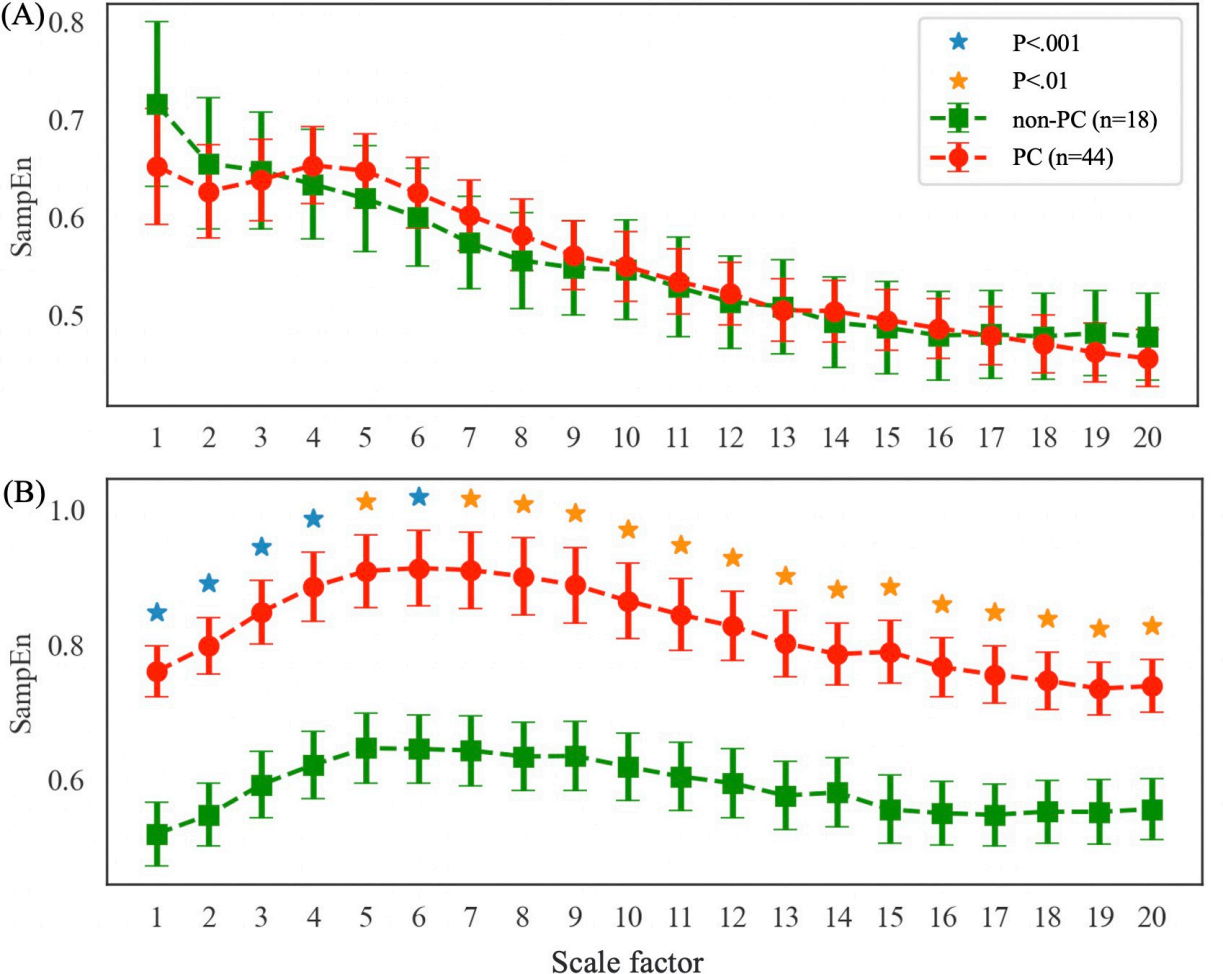
(A) Multiscale entropy analysis of breath-to-breath intervals and (B) respiratory amplitude in pulmonary congestion and non–pulmonary congestion groups of heart failure patients. PC: pulmonary congestion.

Table 2 shows the comparison of respiratory parameters between patients with HF with PC and non-PC. With regard to the respiratory rate and time, the PC group had longer mean TE but a higher mean TE_ratio when compared with the non-PC group, as illustrated in Figure 2. No difference was found in the parameters of RA. MSE analysis indicates similar SampEn values of BBI on various time scale factors among the 2 patient groups with HF (Figure 3A). However, the SampEn values of RA across the scale factors are significantly higher in the PC group compared with those without PC, with higher RA area_1_5 and RA area_6_20 (Figure 3B).

**Table 2.** Respiratory patterns between patients with heart failure with and without pulmonary congestion.

Variable	non-PC^a^ (n=18)	PC^b^ (n=44)	*P* value
Respiratory cycle			
Mean BR^c^ (bpm), mean (SD)	20.70 (3.95)	20.21 (3.83)	.66
BR cv^d^ (%), mean (SD)	32.65 (14.42)	34.69 (14.33)	.62
Mean TI^e^ (s), mean (SD)	1.43 (0.20)	1.42 (0.27)	.84
TI cv (%), median (IQR)	56.32 (8.54-82.28)	74.13 (57.06-108.73)	.10
Mean TE^f^ (s), mean (SD)	1.94 (0.34)	2.17 (0.43)	.03
TE cv (%), mean (SD)	75.50 (47.91)	87.04 (47.80)	.40
Mean TE_ratio^g^ (%), mean (SD)	56.40 (3.36)	59.12 (2.94)	.006
TE_ratio cv (%), mean (SD)	18.30 (6.41)	18.33 (5.46)	.99
Respiratory amplitude			
RA^h^ cv (%), mean (SD)	84.13 (34.54)	70.12 (24.41)	.13
R_RSBI^i^ cv (%), median (IQR)	66.89 (50.28-85.28)	73.74 (59.23-109.45)	.17
RII^j^, mean (SD)	0.20 (0.10)	0.21 (0.09)	.86
MSE^k^ parameter, mean (SD)			
BBI^l^ slope_1_5^m^	–0.02 (0.07)	0.00 (0.08)	.25
BBI area_1_5^n^	3.27 (1.33)	3.22 (1.42)	.89
BBI area_6_20^o^	7.75 (3.05)	7.83 (3.21)	.925
RA slope_1_5	0.03 (0.04)	0.04 (0.05)	.65
RA area_1_5	2.93 (1.03)	4.20 (1.52)	<.001
RA area_6_20	8.86 (3.14)	12.28 (4.84)	.002

a non-PC: patients with heart failure without pulmonary congestion.

b PC: patients with heart failure with pulmonary congestion.

c BR: breathing rate.

d cv: coefficient of variation.

e TI: inspiratory time.

f TE: expiratory time.

g TE_ratio: ratio of expiratory time.

h RA: respiratory amplitude.

i R_RSBI: relative rapid-shallow-breathing index.

j RII: respiratory instability index.

k MSE: multiscale entropy.

l BBI: breath-to-breath intervals.

m slope_1_5: slope of the linearly fitted multiscale entropy curve for scales 1 to 5.

n area_1_5: area under the multiscale entropy curves for scales 1 to 5.

o area_6_20: area under the multiscale entropy curves for scales 6 to 20.

### Factors Associated With PC

By univariate logistic regression analysis, only respiratory parameters of mean TE_ratio, RA area_1_5, and RA area_6_20, and clinical parameters of DBP, NYHA IV, and logNT-proBNP (logarithmically transformed N-terminal pro-B-type natriuretic peptide) were associated with PC (*P*<.05) (Table 3). By multivariate logistic regression, the RA area_1_5 and RA area_6_20 were independently related to PC after adjusting for age, sex, DBP, NYHA IV, and logNT-proBNP (Table 4).

**Table 3.** Univariate logistic regression analyzes factors related to pulmonary congestion.

Variable	Odds ratio (95% CI)	*P* value
Clinical Parameter		
Age (y)	1.026 (0.989‐1.065)	.17
Male, (n [%])	1.400 (0.459‐4.266)	.55
Height (m)	0.016 (0.00‐43.528)	.31
Weight (kg)	0.962 (0.923‐1.003)	.07
BMI (kg·m^–2^)	0.901 (0.799‐1.015)	.09
SBP^a^ (mm Hg)	0.976 (0.951‐1.002)	.07
DBP^b^ (mm Hg)	0.932 (0.886‐0.981)	*.*007
NYHA IV*^c^*	3.421 (1.039‐11.262)	.04
logNT-proBNP^d^ (pg·mL^–1^)	11.865 (1.944‐72.400)	.007
Respiratory patterns		
Mean BR^e^ (bpm)	0.967 (0.838‐1.116)	.65
BR cv^f^ (%)	1.011 (0.971‐1.052)	.61
Mean TI^g^ (s)	0.811 (0.089‐7.358)	.85
TI cv (%)	1.010 (0.995‐1.025)	.18
Mean TE^h^ (s)	4.169 (0.966‐18.004)	.06
TE cv (%)	1.005 (0.993‐1.018)	.39
Mean TE_ratio^i^ (%)	1.311 (1.078‐1.594)	*.*007
TE_ratio cv (%)	1.001 (0.908‐1.103)	.99
RA^j^ cv (%)	0.983 (0.964‐1.002)	.08
R_RSBI^k^ cv (%)	1.002 (0.996‐1.009)	.75
RII^l^	1.814 (0.004‐791.254)	.85
BBI^m^ slope_1_5^n^	1.415 (0.788‐2.539)	.25
BBI area_1_5^o^	0.972 (0.653‐1.447)	.89
BBI area_6_20^p^	1.009 (0.846‐1.203)	.93
RA slope_1_5	1.246 (0.657‐2.364)	.50
RA area_1_5	2.076 (1.256‐3.433)	*.*004
RA area_6_20	1.234 (1.044‐1.460)	.01

a SBP: systolic blood pressure.

b DBP: diastolic blood pressure.

c NYHA: New York Heart Association class IV.

d logNT-proBNP: logarithmically transformed N-terminal pro-B-type natriuretic peptide.

e BR: breathing rate.

f cv: coefficient of variation.

g TI: inspiratory time.

h TE: expiratory time.

i TE_ratio: ratio of expiratory time.

j RA: respiratory amplitude.

k R_RSBI: relative rapid-shallow-breathing index.

l RII: respiratory instability index.

m BBI: breath-to-breath intervals.

n slope_1_5: slope of the linearly fitted multiscale entropy curve for scales 1 to 5.

o area_1_5: area under the multiscale entropy curves for scales 1 to 5.

p area_6_20: area under the multiscale entropy curves for scales 6 to 20.

**Table 4.** Adjusted logistic regression analysis odds ratio and 95% CI results.

Model	Mean TE_ratio^a^, OR^b^ (95% CI)	RA^c^ area_1_5^d^, OR (95% CI)	RA area_6_20^e^, OR (95% CI)
Model 1^f^	1.329 (1.081‐1.634)^g^	2.138 (1.265‐3.614)^g^	1.238 (1.037‐1.477)^h^
Model 2^i^	1.259 (1.005‐1.577)^h^	3.311 (1.563‐7.011)^g^	1.337 (1.068‐1.674)^h^
Model 3^j^	1.306 (1.054‐1.618)^h^	2.148 (1.249‐3.692)^g^	1.229 (1.023‐1.478)^h^
Model 4^k^	1.314 (1.05‐1.645)^h^	2.569 (1.340‐4.925)^g^	1.21 (1.001‐1.462)^h^
Model 5^l^	1.226 (0.954‐1.576)	6.787 (1.838‐25.058)^g^	1.276 (1.004‐1.622)^h^

a Mean TE_ratio: mean ratio of expiratory time.

b OR: odds ratio.

c RA: respiratory amplitude.

d area_1_5: the area under the multiscale entropy curves across scales 1‐5.

e area_6_20: the area under the multiscale entropy curves across scales 6‐20.

f Model 1: age, sex.

g *P*<.01.

h *P*<.05.

i Model 2: age, sex, diastolic blood pressure.

j Model 3: age, sex, New York Heart Association IV.

k Model 4: age, sex, logarithmically transformed N-terminal pro-B-type natriuretic peptide.

l Model 5: age, sex, diastolic blood pressure, New York Heart Association IV, logarithmically transformed N-terminal pro-B-type natriuretic peptide.

### The Ability of Respiratory Parameters to Differentiate PC

**Figure 4.**
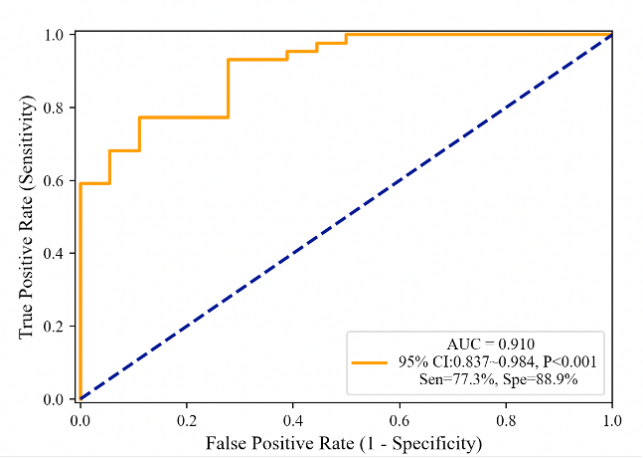
Receiver operating characteristic curve of pulmonary congestion in heart failure identified by multivariate logistic model. AUC: area under the curve; Sen: sensitivity; Spe: specificity.

Through ROC curve analysis, the ability of all relevant indicators to distinguish between participants with and without PC was compared. The AUC for RA area_1_5 was the largest at 0.75 (95% CI 0.63-0.88, *P*=.002, sensitivity of 65.9%, and specificity of 83.3%), as presented in Table 5. Figure 4 displays the ROC curve of a multivariate logistic regression model constructed using combined parameters of DBP, logNT-proBNP, NYHA IV, mean TE_ratio, RA area_1_5, and RA area_6_20, with an AUC of 0.91 (95% CI 0.84-0.98, *P*<.001), sensitivity of 77.3%, and specificity of 88.9%.

**Table 5.** Receiver operating characteristic curves of breathing parameters for distinguishing pulmonary congestion of patients with heart failure.

Variable	AUC^a^ (95% CI)	*P* value	Sensitivity (%)	Specificity (%)
Clinical parameter				
DBP^b^ (mm Hg)	0.282 (0.143‐0.421)	.007	77.8	61.4
logNT-proBNP^c^ (pg·mL^–1^)	0.713 (0.565‐0.862)	.009	88.6	50
NYHA IV^d^	0.645 (0.496‐0.785)	.08	56.8	72.2
Respiratory patterns				
Mean TE_ratio^e^	0.725 (0.590‐0.860)	.006	88.6	50
RA^f^ area_1_5^g^	0.754 (0.632‐0.876)	.002	65.9	83.3
RA area_6_20^h^	0.707 (0.574‐0.840)	.01	52.3	83.3

a AUC: area under the curve.

b DBP: diastolic blood pressure.

c logNT-proBNP: logarithmically transformed N-terminal pro-B-type natriuretic peptide.

d NYHA: New York Heart Association class IV.

e TI_ratio: the mean ratio of expiratory time

f RA: respiratory amplitude.

garea_1_5: the area under the multiscale entropy curves across scales 1‐5.

h RA area_6_20: the area under the multiscale entropy curves across scales 6‐20.

## Discussion

### Principal Findings

In the current observational exploratory study, we demonstrated the feasibility and capability of a wearable device (“SensEcho” by Beijing SensEcho Science & Technology Co) to differentiate PC severity by detecting nocturnal respiratory signals (cycle and amplitude) in hospitalized patients with HF. First, we found the hospitalized patients with HF with PC had a longer mean TE and subsequently an increased TE_ratio than those without PC. Second, by using the MSE algorithm to assess respiratory complexity, the increased RA complexity could independently indicate a PC condition.

### Prolonged Expiratory Time in Patients With PC

It was well-known that patients with HF showed increased rest respiratory rate and decreased amplitude than healthy controls, characterized as a “rapid and shallow” pattern. However, our study found that these parameters about respiratory rate and amplitude recorded at nighttime failed to differentiate patients with HF with and without PC. Unexpectedly, patients with PC were found to show a longer expiratory time and a ratio, which is commonly seen in patients with asthma or chronic pulmonary disease who had increased airway resistance but seldom reported for patients with HF. Theoretically, a prolonged expiratory time is reasonable and seen in patients with HF and PC. At first, the PC-induced airway compression or bronchial congestion–induced mucosal edema could result in bronchial flow limitation, increased ventilation-perfusion mismatch, and increased work of breathing [26]. In addition, experimental studies have shown fluid overload leads to a decrease of the diameter of both small and large airways [27]. All these factors could contribute to the prolonged expiratory time as seen in patients with obstructive lung disease [28]. Second, ventilation at rest requires only the inspiratory muscles, but the expiratory is a passive process in normal conditions. When with disease or increased ventilatory demands, the expiratory muscles begin to play a role. For heart failure, Zoccal and Machado [29] found that volume overload in rats with HF displayed active expiration and the recruitment of abdominal muscles. Furthermore, enhanced central chemoreflex gain was observed in the volume overload HF model and played a role in the development of respiratory dysfunction. After establishing a volume-overload HF model in rats via an arteriovenous fistula, episodic hypercapnic stimulation could further increase active expiration [30]. Taken together, prolonged TE or an elevated expiratory ratio may serve as promising indicators of HF-induced PC. However, these findings should be interpreted with caution in patients with HF with coexisting obstructive lung disease (eg, COPD or asthma)—a population excluded from this study. Such comorbidities share overlapping pathophysiological mechanisms that alter breathing patterns, including bronchial flow limitation, increased ventilation-perfusion mismatch, and elevated work of breathing. Thus, the specificity of these respiratory metrics for PC detection could be compromised in patients with concurrent airflow obstruction.

### The Respiratory Complexity Assessed by MSE

Periodic breath is a consequence of respiratory control system instability and is well-recognized in patients with HF due to prolonged circulatory time, increased carbon dioxide chemosensitivity, lower carbon dioxide setpoint, activation of pulmonary C-fibers due to congestion, increased sympathetic nerve activity, or modulation of the ergoreflex, etc [26]. It is characterized by waxing and waning of tidal volume with or without interposed apnea, which contributes to the breath pattern (time, rate, and amplitude) complexity. Recognizing the complexity of periodic breathing could serve as a prognosticator. For instance, Asanoi et al [31] calculated the respiratory instability based on the frequency distribution of the respiratory spectral components and the ultra-low frequency component and found that it has prognostic significance independent of sympathetic activation. Furthermore, respiratory complexity is also closely related to disease severity, a valuable guide in the management and treatment of patients with HF. Similarly, Corrà et al [32] assessed cyclic fluctuations in minute ventilation and found that patients with higher variability in respiratory rate had worse clinical status and exercise capacity.

### Clinical Perspectives and Future Directions

For the first time, this study demonstrates the feasibility of using wearable devices to continuously monitor respiratory signals in patients with HF, coupled with MSE analysis—a robust method for quantifying physiologic time-series complexity [33-35]. Our findings reveal that elevated MSE amplitude, reflecting increased respiratory complexity, is strongly associated with PC. Given that worsening PC drives HF exacerbations and hospitalizations, this home-based MSE amplitude monitoring approach may offer a novel, noninvasive tool for early detection of worsening PC, enabling timely interventions to reduce rehospitalization rates [36]. However, several critical steps are needed to translate these findings into clinical practice. First, prospective validation in larger, diverse HF cohorts (eg, across NYHA classes, etiologies, and comorbidities) is essential to confirm generalizability. Notably, future studies should include patients with concurrent conditions like COPD or obesity, which may confound respiratory signal interpretation. Second, longitudinal studies should assess whether day-to-day MSE amplitude fluctuations precede symptomatic HF deterioration and set a rule to alert, potentially serving as a predictive biomarker. Third, seamless integration of wearable-derived MSE data into electronic health records and telehealth platforms, leveraging machine learning models to analyze MSE patterns alongside multimodal data (eg, daily vital signs and weight trends) for enhanced exacerbation prediction and risk stratification.

### Limitations

This study has several limitations that warrant consideration. First, as an exploratory investigation, our findings are derived from a small, single-center cohort, which inherently limits the generalizability of the results. External validation in larger, multicenter populations—particularly those encompassing broader demographic and clinical spectra—is necessary to confirm the robustness of a respiratory complexity biomarker in HF. Second, we excluded patients with concurrent pulmonary diseases to isolate HF-specific respiratory patterns. Consequently, the applicability of wearable device–based respiratory pattern analysis in patients with HF with comorbid pulmonary conditions remains uncertain. Third, to mitigate potential confounders from daytime physical activity, our analysis focused exclusively on nocturnal respiratory signals. While this approach enhances signal purity, it precludes assessment of whether daytime respiratory patterns—which may capture dynamic responses to exertion or postural changes—hold superior predictive value for HF exacerbations. Future studies should evaluate 24-hour respiratory monitoring to determine the optimal sampling window for clinical prediction.

### Conclusions

In this small-scale exploratory study, wearable-based MSE analysis successfully distinguished between hospitalized patients with HF with and without PC, as evidenced by prolonged expiratory phases and increased RA complexity in the PC group.

## Abbreviations

area_1_5: area under the curve of multiscale entropy for scales 1 to 5
area_6_20: area under the curve of multiscale entropy for scales 6 to 20
AUC: area under the curve
BBI: breath-to-breath intervals
BR: breathing rate
COPD: chronic obstructive pulmonary disease
DBP: diastolic blood pressure
EF: ejection fraction
HF: heart failure
logNT-proBNP: logarithmically transformed N-terminal pro-B-type natriuretic peptide
LUS: lung ultrasound
MSE: multiscale entropy
NYHA: New York Heart Association
PC: pulmonary congestion
R_RSBI: relative rapid-shallow-breathing index
RA: respiratory amplitude
ROC: receiver operating characteristic
SampEn: sample entropy
TE: expiratory time
TE_ratio: ratio of expiratory time
